# Topics of Public Interest

**Published:** 1908-02

**Authors:** 


### TOPICS OF PUBLIC INTEREST.

### Education in England.

**Ambassador Reid's Address to New York Teachers, at Syracuse.**

[*New York Tribune*, December 27, 1907.]

It has been represented to me that this body of practical and prominent teachers of the Empire State would be interested in some account of what schools and teachers in England are doing.

. . . . . . . . . . . . .

I shall speak to you, then, briefly, of some details of past and present English educational work. But I shall do no violence to the maxims either of Dogberry or Don Quixote; shall enter upon no comparisons with our own work in similar fields. There are two reasons. First, all comparisons between countries are apt to be odious. Second, unless far more time were taken than is at our disposal to-night, for a careful statement of varying circumstances, all comparisons are sure to be unfair.

In any consideration of English education for the masses, it must be remembered that a National system for it did not exist before 1870, and could not be said to have reached good working order before 1892. The Government gave no assistance whatever for elementary schools (i. e., for what we should call common schools, or primary schools) until 1834, when the House of Commons made its first appropriation of £20 000. This was to be used solely for new school buildings. Not till 1839 did the Government make any appropriation at all for more direct aid to popular education.

. . . . . . . . . . . . .

Until William E. Forster in 1870 carried through the bill to provide for public elementary education in England and Wales, the Government itself could hardly be said to have taken much share in real educational provision for the poorer classes, and not a great deal even for the middle classes. Nevertheless, such as their system was, and for what it undertook, it had long been of rare excellence. It had admirably accomplished—for a certain number—the highest aim of education; it had been a wonderful developer of character. Public schools, Eton and Harrow, Winchester and Rugby, and many another leading up to and co-operating with the two Universities, had been such a nursery of statesmen, of soldiers and sailors and great proconsuls and civil administrators throughout the Empire on which the sun never sets, as the world had never before seen. It may have been a fanciful notion, attributed to the Iron Duke, that Waterloo was won at Eton, but certainly the secret of Anglo-Saxon superiority in the 17th and 18th centuries was largely to be found in the British schools and universities.

FORMER CONDITIONS IN ENGLAND.

The secret of some other things was to be found in the chaotic and undeveloped state of popular elementary education. The long reign of Queen Victoria had but recently begun, when in February, 1839, Lord John Russell wrote to the Lord Lansdowne of the day: "I have received Her Majesty's commands to make a communication to your Lordship on a subject of the greatest importance. Her Majesty has observed with deep concern the want of instruction which is still observable among the poorer classes of her subjects. All the inquiries which have been made show a deficiency in the general education of the people, which is not in accordance with the character of a civilised and Christian nation." Continuing to speak for Her Majesty, Lord John went on to specify a lack of qualified teachers, imperfect teaching, deficient inspection of the work done by the schools of both the established church and the Non-Conformists, and finally of the neglect of the subject by Parliament.

Four years later, inspectors reported that the teaching in these schools was so bad that only half the scholars learned to read and only a quarter of them to write. And four years after that, now almost in the middle of the 19th century, Macaulay, in a speech in the House of Commons, gave the reason: "How many of these teachers," he said, "are the refuse of other callings, discarded servants or ruined tradesmen, who cannot do a sum of three; who would not be able to write a common letter; who do not know whether the earth is a cube or a sphere; and cannot tell whether Jerusalem is in Asia or America; whom no gentleman would trust with the key of his cellar, and no tradesman would send of a message."

Even as late as 1861, about the time our Civil War broke out, the Newcastle Commission reported almost as unsatisfactory a state of affairs. It considered that only about one-fourth of the children in the schools got a tolerable facility in reading, writing and arithmetic,—the great majority leaving school between the ages of ten and eleven. It told of a public school with such primitive facilities that, when the writing lesson was given, four boys were required to carry ink bottles up and down between the desks, so that each boy in turn might dip his pen in the ink. And finally this Commission said concerning the private school teachers in one part of London: "None are too old, too poor, too ignorant, too feeble, too sickly, too unqualified to regard themselves and to be regarded as fit for school-keeping. Domestic servants out of place, discharged bar-maids, vendors of toys and lollipops, keepers of small eating-houses, of mangles or of small lodging houses, needle women who take in plain or slop work, milliners, consumptive patients in an advanced stage, cripples almost bed-ridden, persons of at least doubtful temperance, outdoor paupers, men and women of seventy or eighty years of age, persons who spell badly, who scarcely write and who cannot cipher at all—such are some of the teachers, not in remote rural districts but in the heart of London." In recalling this and other accounts of the time it is well for you to bear in mind that in all countries reformers have sharp voices and use many staccato notes.

But Matthew Arnold was not of that class: yet he reported in 1869 that nearly half the children he examined had been less than one year at school, and half the rest less than two years.

Now, to end this statement of earlier conditions, which has been really necessary to a comprehension of the present situation, it should be added that the schools thus described might be either purely private enterprises, sometimes aided a little by local taxation, or might be under the management either of the established church of England, or of the British and Foreign School Society, representing the bulk of the Non-Conformist churches, or of sundry minor religious organisations. By far the greater number were under some distinct and positive sectarian control. Great sums had been invested by the different denominations, in school buildings and in supporting schools, when there was little other support for them. Their work had come gradually to be supplemented not only by fees but by allowances from the local taxation, and finally from the Government. Thus the churches controlled the schools; the local taxpayers had pecuniary interest in them, the parents who paid fees had, and finally the general Government had.

As would be naturally inferred, the churches that built them up insisted on religious teaching. In the case of the established church this meant the Bible, Church hymns, the Church Catechism, and particularly the doctrine of the Trinity; and at first pupils coming into such a school from Non-Conformist families or Agnostic or Jewish families, or from aggressive unbelievers, had to receive the same instruction. Here, of course, was one opening for trouble; and another was to be found among local taxpayers, not connected with the Church of England, or perhaps with any church. With Non-Conformist schools the difficulty was somewhat different. They were disposed to be content with what was known as Cowper-Temple teaching; i. e., as legally defined in the Act of 1870, without "religious catechism or religious formulary, distinctive of any particular denomination." Subject to that restriction, whatever religious instruction the local authorities desired could be given. This Cowper-Temple teaching, though apt to be satisfactory to the majority of Non-Conformists, did not satisfy the established church, or the unbelievers, and might not always satisfy the local taxpayers. As a matter of fact, however, it evoked little protest, excepting from the established church.

Now it is easy for an American to say that all this confusion and dissatisfaction could be avoided by confining the public schools to secular instruction, and leaving religious training to the Church and the family. But it is not so easy to show how vested rights, going back often for a century or more, can thus be preserved; nor is it easy to show how the churches, which invested and were encouraged to invest their money and labors for one purpose, are to be reconciled to the arbitrary diversion of their investment, long afterwards, to another purpose. Between 1869 and 1876 houses for over a million school children were erected by denominational agencies, and the total of voluntary subscriptions for that purpose in that time was over £3,000,000. Besides the claim in equity which on the basis of such facts the churches assert, it is probably true that the majority of the English people, however much they may differ as to details, and to whatever rival sect they belong, would be still more discontented if all religious teaching were to disappear from their schools. There is increasing impatience, no doubt, with the conflicting demands and disputes of the churches, a growing tendency to say "a plague on both your houses; let the tax-paid education be purely secular"; but in spite of such outbursts, I believe the decided majority of the taxpayers still think religious instruction a necessity for the rising generation, and do not think they would have adequate security for getting it, if it were excluded from the tax-supported schools. Until 1870 the daily reading of the Bible was an essential condition of getting any Government aid for an elementary school, and it is still habitually read in most of the schools, even where not required by any authority.

. . . . . . . . . . . . .

SOME ERRORS CORRECTED.

During the popular debates over this measure, I received a letter from the Editor of "The Salisbury Times," besides several from private sources, all calling my attention to a startling statement made in a speech on the subject at a political meeting in Salisbury, by a well-known and perfectly reputable Conservative candidate, to this effect:

"In Australia, since religious teaching was abolished in the day schools, crime has increased 75 per cent. In the United States in 1850 there was one crime to every 3,422 of the population, but to-day there is one criminal for every 300. In Denver, out of 10,000 boys, 2,000 of them have been in jail. Now, we do not want the same thing to happen in Great Britain."

I was asked if these statements were not misleading, and I prepared such a reply as careful inquiry seemed to show that the facts warranted. But there was at the moment no such storm-centre in British politics as this religious phase of the educational question; and on second thoughts it appeared wiser for a diplomat to obey the old rule about rigorous care to avoid getting in any way involved in the domestic debates of the country to which he was accredited,—even if it should be at the temporary cost of not promptly correcting misapprehensions about his own country.

Now, it would have been easy, first, to call attention to the curious fact that the statements were strikingly like some unwise stories published from time to time, some only a few years earlier, in American reviews of high standing, concerning an alleged increase of juvenile crime in London, following the extension there of the free school system. Next, as to the allegations concerning the United States, it might have been said at once that they were inexact, and that, even if they had been accurate, they would have needed to be made more complete to avoid giving an inaccurate impression.

They were inexact because the latest census statistics available, those furnished by the Census Office in 1904, show that instead of one criminal to every three hundred of population, there is only one to every 990; also that there has been a reduction between 1890 and 1904, not merely in the proportion of criminals to total population, but also in the actual number of criminals in spite of the increase of population; and finally that the Census Office believes that its own returns of criminals before 1880 were imperfect, making the number previous to that date too small, and consequently exaggerating the increase in the next decades.

. . . . . . . . . . . . . . .

SUGGESTIVE FACTS FOR AMERICANS.

And yet I cannot help feeling that on the general subject we might profitably take a hint from the old country. Whatever else we may say about the English schools they do turn out well behaved, orderly boys and girls, respectful to those set over them, grounded in the morals of Christian civilisation, with an instinctive sense of obedience to law and a becoming regard for the authorities that represent it. Would we be any the worse off if we had more of these qualities here? May it not happen that in our effort to keep all questions of religion and morals in what we consider their proper place, they may in reality be left without any place in the training of a good many children? If the interest of the Republic requires that every child should be compelled to learn to read its laws, does not the same interest as imperatively require that every child should be taught, and should be unable to escape being taught, the absolute necessity of respect for those laws and of prompt and dutiful obedience to the officers of the law? Does not the interest of the Republic further demand that the coming citizens shall have some idea of our old beliefs in the Fatherhood of God and the brotherhood of man, or at least shall be thoroughly grounded in the great principles of the moral law, without which neither ordered liberty nor civilisation itself can exist?

If English schools, according to our ideas, go too far, in teaching creeds, may we not be going too far the other way, in some parts of the country at least, in excluding altogether, or in giving too little space to teaching unsectarian religion and morals, to enforcing respect for authority, and to training the habit of mind that secures unhesitating obedience to law, and to its officers? In London the policeman, the representative of law, often controls the biggest and angriest crowd by lifting his hand, in cases where the New York policeman has to lift his club. Nay, here the giddy chauffeur, for a single example out of many, gayly snaps his fingers at the uplifted club, and has to be run down on a motorcycle. Even then, when caught, he is apt to tell the presumptuous policeman he means to have him "broken" for his pains. Such a threat in London would railroad him to a long term in jail. The mere failure to stop, the moment a policeman lifts his hand, is generally in England unthinkable; the imagination is staggered to conceive the punishment that might befall the insensate and foolhardy person who would venture on such unprecedented lawlessness. Some cause has produced this difference. Is it improbable that early training in a school that could be nowise escaped by the growing boy had something to do with it?

. . . . . . . . . . . . .

FRILLS, ESSENTIALS AND DISCIPLINE.

American critics of tendencies in their own schools sometimes object to the "fads and frills" which, as they say, keep the children from learning "the three R's." It will be observed that the London elementary schools likewise provide for a good many so-called "frills." But it must be noted that these are not permitted to take the place of the essentials. Whatever else a London child may learn at a "provided school," he must and does learn to read, write and cipher. Two out of the three at least he generally learns remarkably well. Nothing is apt to strike an American more, when he comes to know the product of English elementary schools, than their thoroughness in these essentials. I have rarely seen a domestic servant who did not have a fairly good handwriting, spell with more accuracy than some of our own misguided college professors, and compose a clear letter, well-expressed, in civil phrases, not offensive by an unwarranted familiarity or wanton assurance in demanding the time of a stranger, not verbose or slangy; in fact likely, by its appearance and manner at least, to create a good impression. Would that we could say as much for all the graduates of our colleges.

In most of the London schools there are three departments, those for boys, girls and infants. An average number for the three would be about 1,000. There are also schools in which the sexes are not separated. About half the teachers in 1869 were women and girls, by 1900 they had become three-fourths. Certified masters of schools are paid about £129 per annum, say $640.00; and certified mistresses about two-thirds as much. Pupil teachers are put in training, on application and favorable reports, at 14 years of age; and after a year, study only half the day, teach the other half and are paid a graded salary which at the end of three years more, rises to £30 for boys and £24 for girls. Women are eligible for Educational Committees, and their service seems to be popular.

The general limit for compulsory attendance at elementary schools was thirteen years, but the local authorities now have the power to raise it to fourteen, and the prevailing tendency is towards an exercise of this power. The penalty on parents for neglect is £1 with costs. The pupils are graded by various standards, known as standard 1, the lowest, and so on up to standards 5 and 6, which represent the highest elementary work, and standard 7, which denotes the distinct extension of the work into the secondary field.

Discipline in the schools is generally very well maintained; pupils of both sexes are early taught obedience, courtesy and respect—sometimes even yet in the old way! Persuasion and kindness are first tried; the effort is to lead the pupil by rewards rather than by punishments. But the hard-headed local authorities have generally not the remotest intention of spoiling the child in order to spare the rod, and the traditional cane is still served out to the head masters and the head mistresses along with the other school supplies. It is not often used, and never without care and some thought of possible legal reprisals, but it is there and it is used if needs must. Perhaps the lad's opinion of Archbishop Temple, at Rugby, may be taken as the ordinary schoolboy's general notion about this application of discipline, when it does come: "He's a beast, but a just beast."

There is a marked tendency in most of the elementary schools to freshen the work, take it away from the old routine methods and make it a real process of drawing out the latent capacities of the child and encouraging it to think, to feel its own way, and to learn for itself. There are many illustrations and experiments, occasional excursions and object lessons. Efforts are made to use the successes of pupils as an abiding stimulus for the schools, and the permanent tablet on the wall serving as an "honor board" is a frequent feature. The local authorities sometimes offer a valuable picture as a prize to a class or a school that in some way distinguishes itself, and with a thrift almost Yankee in its subtlety gain by what they give, since the picture remains as the permanent adornment of the schoolhouse!

### ENGLISH SECONDARY EDUCATION.

In 1861 Matthew Arnold, after inspecting foreign school systems, returned to report to the Royal Commission on Endowed Schools, which had sent him out, with the appeal: "Organise your secondary and your superior education." Ten years later Professor Huxley, in the first London School Board, urged an arrangement by which a passage could be secured for children of superior ability from the elementary schools to schools in which they could obtain a higher instruction. No educational system, he said,—in a notable speech, now familiar, I think, to most American educators—no such system would be worthy the name of a National system, "unless it established a great educational ladder, the bottom of which should be in the gutter and the top in the University, on which every child who had the strength to climb might, by using that strength, reach the place for which nature intended him."

But in 1907 the appeal of Matthew Arnold is not yet fully answered, the dream of Professor Huxley not yet fully realised. Unsystematic as the primary education has been found, secondary education is still more so. There are in London "Higher Grade Schools," "Organised Science Schools," and "Higher Elementary Schools." Some of these are merely the highest class of elementary schools, reaching up into subjects proper to the first years in secondary education; some others represent a rather confused effort to promote secondary education, technical education and commercial art education side by side. Some of them give efficient instruction in chemistry, physics, electricity, physiology, botany, French, German, algebra, geometry, trigonometry, English literature and history. It is not clear that many of them enable their students to pass on to universities. A "Higher Grade" school at Leeds has a superior record in that respect, ninety-three of its pupils having matriculated at London University, and sixty-five having taken university degrees.

There is another development of secondary education directly from the elementary schools, generally more practical in its nature, and tending often to scientific or technical courses. This is the one stimulated by a system of scholarships, junior, intermediate and senior, offered by the London County Council and open to competition by the pupils in the elementary schools. About six hundred junior scholarships are thus given in a year to boys and girls under thirteen years of age, and nearly all go to pupils of the Council schools. These keep the children at a Higher Grade Council school or at a secondary school for two years, pay fees where there are any, and give the pupil for his maintenance for the two years an allowance of £20; but the parents of the children receiving them must have an income less than £150, say less than $750. The boy or girl who gains one of these scholarships gets tuition one year beyond the usual fourteen-year limit, and is then able to compete for an intermediate scholarship. These again are open to any under sixteen, whose parents have an income of less than £400 a year; and when won, secure any fees in secondary schools, together with an allowance of £55 for maintenance for two years. There are about one hundred of them a year for all London, and they practically denote the high water mark of Council school education. There are still, however, seven or eight junior scholarships a year, and these carry the successful contestants for three years at a university, with tuition fees and a maintenance of £30 a year. This, it will be observed, constitutes a genuine scheme of state supported secondary education. It is not open to all who may have passed through the lower classes and feel like keeping on. But it is open to the selected few who have shown special qualifications for a higher training, and whose parents are poor; and to these most hopeful children of the Empire their government extends not merely free tuition but free support. Those seeking the old universities, and many of those seeking scientific courses at the new ones, still resort, if they can, either to schools conducted for private profit, or to the public schools, so called, i. e., endowed schools like Harrow, Rugby, Westminster, St. Paul's, Manchester Grammar School and thirty or thirty-five more. Many of these are ancient foundations, and they have borne a vital relation to some of the proudest pages of English History. At least two of them, Winchester and Eton, were well endowed for the time and in successful operation before the discovery of America. A much larger number were established before the Colonies at Jamestown and Plymouth were; and most of the more noted ones before our Declaration of Independence. These schools belong therefore to our history, too. They recall to us as well as to Englishmen, in their scrupulously guarded rolls, their successive generations of eminent men. They make alive again the proud records above the sacred dust of myriads of the great departed all over the land, from stately cathedrals to the quiet churchyard of the remotest hamlet. This sacred dust it was that gave the inspiration to Oliver Wendell Holmes's eulogy of England and her illustrious dead, and justified his vivid outburst:

> One-half her soil has walked the rest,
> In poets, heroes, martyrs, sages.

These public schools are in general splendidly healthy and useful yet; within their field and for their purposes unsurpassed in the educational work of the world. But their field, until recent years, has been almost exclusively the Humanities; and their aim, Senior Wranglerships and double firsts in the universities, the front bench in the House of Commons, and responsible places all around the world in the administration of the Empire, or in their most esteemed services, the Army, the Navy and the Church. Till 1851 mathematics was not compulsory at Eton, nor French till 1862. Natural Science was scarcely noticed.

### CO-ORDINATING THE SCHOOLS.

An English educational writer has unfairly said that "England is the country where dead systems live." A student of her educational history might be tempted to accept that judgment if he looked merely to the fact that it was only as late as 1895, and after the notable report of Mr. James Bryce (now His Majesty's Ambassador to the United States) on the best methods of establishing a well organised system of Secondary Education in England, that a central organisation was created to co-ordinate all these previous divergent and unregulated schools which furnish the links between the elementary schools below and the universities above, as well as the technical and scientific schools that ought to be above. Before that date the most considerable part of the secondary education work was under the control of the Charity Commission. The Science and Art Department had been administering the newer plans to meet the special demand for technical instruction and had the disposition of an income for this purpose of nearly a million pounds (five million dollars) per annum. The Education Department had charge of the elementary schools, and, as has been seen, had developed from these some interesting advance into the secondary field. At last in 1900 a Board of Education was created, which took over the secondary educational work of the Charity Commission, of the Science and Art Department and of the Education Department.

The work thus at last co-ordinated had reached great proportions. In 1892 the Charity Commissioners reported the educational endowments in England alone, available for secondary education, as producing an income of over £697,000 a year, say three and a half million dollars,—not to reckon at all the value of their buildings and sites. In 1897 the Education Department made a census of English Secondary Schools. Its returns were thought to be vitiated by including many not really entitled to rank as secondary schools; but it reported 6,209 of them, with pupils numbering almost ten in the thousand of the whole population. The Science and Art Department received the customs and excise money (popularly "the whisky money"), and from this fund technical schools were given nearly £864,000 in 1900, while the sum raised for the same purpose by rates (local taxes) amounted to £106,000 more, say in all over four and three-quarter million dollars. Under the latest legislation this goes to the County Councils, and the Councils of County boroughs and of urban districts. It must be spent on secondary education. They have authority to raise more by local rates, but this in the case of Counties must not exceed a two-pence rate.

At present the regulations forbid teaching more than thirty-five scholars together at one time. They permit fees that may be approved by the Board, but require that one-fourth of the school places be open without fees to pupils from elementary schools, who pass a satisfactory entrance examination. The number of such schools in England and Wales recognised by the Board and given State aid was 689, in the years 1905-'06, and the number of pupils was 94,689.

. . . . . . . . . . . .

Attendance at English elementary and secondary schools is still apt to stop at the age of fourteen, if not earlier, but the tendency begins to be toward a longer stay. Sports are still an absorbing part of the school work, and interest in them is almost as necessary for the teacher as scholarship. The teachers are not so apt to show individuality and energy as they are to be careful and pertinacious. Great attention has been paid to the training of teachers of late years, but the system of "pupil teachers" has still to eke out the supply. In the great cities there is an enormous and interesting development of evening schools. Trade schools are increasingly numerous and popular. In the great technical schools there is a noticeable absence of pupils who seek easy electives, and are there chiefly for the degree. Their work is often not very rapid, but it is apt to be thorough. In all these directions the admonition of the Prince of Wales on his return from his Eastern trip has been heard, and England has "waked up."

It will have been noted that in elementary schools the prevailing tendency of late years has been toward sense training, object lessons and manual employment. So among secondary schools the tendency has been toward studies fitting for practical scientific, or manufacturing and commercial life. Both are more democratic than the historic public schools; and there begins to be a greater mingling of classes in the more recent secondary schools, in the scientific technological schools, and in the newer universities to which they lead.

UNIVERSITIES AND RHODES SCHOLARS.

Naturally, then, the chief new development of educational activity has been in the expansion or creation of advanced institutions to carry on this practical training beyond the secondary stage. Until less than a century ago, there were only two Universities in England and Wales. Now there are ten. Practically all the new ones yield the preëminence in the old classical, mathematical, and philosophic training to Oxford and Cambridge, while they strive to occupy more thoroughly the undeveloped field of scientific and technological work. Then there are twenty-three Technical Institutions in England and Wales, recognised by the Board of Education, and 231 schools of art applied to the industries.

The Universities have been slowly led to examinations for the various kinds of secondary schools, some of which serve as leaving examinations for the schools and others as matriculation examinations for the universities, though often used by the recipients for other purposes. Oxford and Cambridge took up this work near the middle of the last century, first separately, then in a joint board. Subsequently London University undertook it on a large scale, and Durham, Victoria and Birmingham have moved in the same direction. The City and Guilds of London Institute also held examinations for technical schools and classes throughout the country.

University work proper is beyond the limits of what you are considering at these meetings; but a word in closing might be given to the Rhodes scholarships at Oxford. We have almost a hundred young American graduates there, distributed through the colleges of that venerable and illustrious University. They are chosen on examination, two from each State and Territory; they are given free the best the university can offer through a three years' stay, and they receive from the fund an allowance of £300, say $1,500, per year for their maintenance. The purpose of the great man who founded this trust was to increase intimate and friendly relations between the most highly educated classes of the Mother country and those of her "giant offspring of the West"; and to further a good understanding between the three nationalities included in the arrangement, England, Germany and the United States. I have met with these Rhodes scholars at their annual reunion at Oxford; and I am glad to testify here at home to their admirable appearance and conduct, and to the favorable opinions of them expressed to me by the Oxford Dons with whom I conversed. As one saw them together, breaking in upon the cloistered quiet of those historic halls, he might almost imagine himself at a big middle-West College in our own country. He would scarcely be able to single out the German Rhodes scholars from the rest, and quite unable to tell Americans from Australians or Rhodesians or Newfoundlanders or Cape Colonists or New Zealanders. But about them all was the air of new worlds and a new era. One might almost fancy their eyes had already seen the glory of the time when under the leadership of the English-speaking peoples the war-drum throbbed no longer, and the battle flags were furled, in the parliament of man, the federation of the world.

---

### U. S. Navy Medical Corps Gets a Stimulus.

### Men in Training Inspired With New Hope by President Roosevelt's Policy.

By WALDON FAWCETT

[*New York Tribune*, January 12, 1908.]

THE action of President Roosevelt in detailing Surgeon Charles F. Stokes, U. S. N., to command the United States hospital ship Relief has stimulated ambition among officers of the medical corps of the navy. The President's vote of confidence in the naval surgeons in the face of the resignation of Rear Admiral Brownson and the protest of line officers of the navy has inspired the medical men with new hopes, and their influence is already manifest at the United States Naval Medical School at Washington, where the officers of the medical corps are trained for their diverse duties on shipboard and at the various shore stations. Heretofore only a small percentage of the graduates of American medical colleges has been attracted to the naval service, and the present class at Uncle Sam's school for navy surgeons numbers only about thirty-five young men, but a largely increased enrolment is now expected.

With the Republic rapidly adding to its roster of fighting ships, a hospital ship being placed in commission and long cruises becoming the order of the day, there has come imperative need for an expansion of the medical corps. How to accomplish this has been puzzling the officials of the bureau of medicine and surgery at the Navy Department for some time. It is small wonder, then, that they have hailed with delight the added prestige that has come to the corps, confident that it will attract to the service many young physicians now on the threshholds of their careers, and affording just the sort of material desired for building up the personnel.

While the Naval Medical School is not, of course, so large or so pretentious an institution as the United States Naval Academy at Annapolis, where the line officers are trained, it is nevertheless an interesting seat of learning and one which is in all respects creditable to the nation. Considering the short time it has been in operation—not quite six years—the development has been rather remarkable. The need for an institution where a systematic course of instruction could be provided for officers of the medical corps of both the army and the navy was recognised from the early days of the government, but despite frequent agitation on the subject the naval officials were not able to accomplish anything in this direction until 1893.

In that year Surgeon General Tryon started a "department of instruction" at the United States laboratory in New York City, and the results were satisfactory, but the conditions resulting from the Spanish-American War made it impossible to continue the project. In 1902, however, Surgeon General Rixey, the present head of the medical corps, took hold of the matter in earnest and procured the establishment at the national capital of the United States Naval Medical School and Hospital.

As a site for this college for navy surgeons he obtained the buildings and grounds of the old naval observatory, in the western part of the city of Washington and on a knoll overlooking the Potomac River and a large part of the capital city. The old observatory building, a rambling, spacious, white structure, surmounted by the now unused observatory dome, made, when remodelled, an excellent nucleus for the home of the new school. To it have been added supplementary structures, including power plant, laundry, stables and so forth, and at last a new hospital, completed only about a year ago. What has been done is only a beginning. A plan has been prepared providing for new buildings for officers' quarters, for an enlargement of the hospital to a capacity of five hundred beds, and for other new construction that will enable the headquarters of the navy medical corps to come at least a little nearer taking rank with the new home of the Naval Academy at Annapolis and the rejuvenated home of the Military Academy at West Point.

The student officers of the medical corps, or assistant surgeons, as they are officially designated, enter the Naval Medical School immediately upon their appointment to the service, and each newcomer in the corps spends at least half a year in this institution, gaining the advantages of what is virtually a post-graduate course that supplements the instruction previously received at some college of medicine. The six months' course prepares the young surgeon for any duty which may thereafter fall to his lot afloat or ashore.

The course of study is essentially practical and includes tropical medicine, bacteriology and laboratory work, naval surgery, drills and so forth. Indeed, there are few graduates of our best medical schools who are afforded an opportunity equal to that of the newly commissioned assistant surgeons of the navy for rounding out their education as physicians and surgeons; and all the while these newly enrolled officers are receiving the full pay of their rank, with allowances.

To the average layman, who may imagine that the qualifications of a navy surgeon consist only of a technical knowledge of pills and powders, it would prove a revelation to visit the Naval Medical School during the morning hours on any week-day. Promptly at 9 o'clock he would observe all the student officers emerge from the building and for a considerable interval devote themselves to a strenuous physical programme, including "setting up" drills and winding up with a brisk run of from half a mile to a mile. Then follows a litter drill in the open air, which tends to give the young men the utmost facility in the quick-handling of stretchers. After a change to regulation uniforms the young officers devote a quarter of an hour or more to the sword drill, and at times this is followed by a period of practice in wigwag signalling. Having aroused all their faculties by this physical activity, the assistant surgeons next put on flowing black robes and plunge into laboratory work. Lectures by eminent specialists and practical work in the hospital are also features of this valuable initiation into the naval service.

As has been said, the student officers are on Uncle Sam's payroll from the day they enter the medical school. A physician enters the service as assistant surgeon, a grade which corresponds in rank to that of lieutenant, junior grade, in the navy, and to that of first lieutenant in the army, and carries pay at the rate of $1,500 a year. In addition, quarters are provided, or an allow ance of $228 a year is made, where quarters are not available. Three years from the date of his first commission the assistant surgeon is entitled by law to promotion to a grade corresponding to that of lieutenant in the navy or captain in the army, and this means, of course, an advance in pay and allowances. Further promotions come as vacancies occur until the medical corps officer reaches a grade equivalent in rank to that of captain in the navy or colonel in the army, with a minimum salary of $3,500 and allowances.

All army and navy officers under the rank of rear admiral receive 10 per cent. additional pay for every five years' service until such bonus reaches 40 per cent; and here the officer of the medical corps has a distinct advantage over the officer of the line; for when a physician is commissioned as assistant surgeon he is given credit for five years' constructive service—the government in this way recognising the cost in time and money of his professional education. Thus the pay of an assistant surgeon is really $1,650 a year. The highest pay of a medical director, the highest grade in the corps, is $4,500. The surgeon general receives the rank, pay and allowances of a rear admiral.

Although the naval medical school has so far cost the country a comparatively modest sum, its equipment is noteworthy in many respects. It has a handsome lecture room, hung with charts and provided with other appropriate accessories; a library stocked with a superior collection of technical books, a great variety of instruments, some of rare value and all of importance, among them a number lent to the school by Surgeon General Rixey; special apparatus in the way of stretchers, signalling outfits and so forth, and a moving platform that imitates the motion of a ship's deck, for stretcher practice under sea conditions.

Not only do the newly enrolled assistant surgeons receive training at the naval medical school, but there is also a course of instruction for passed assistant surgeons and surgeons—veterans in the service—who are detailed for study from their ships or from some of the seventeen naval hospitals in various parts of the country, in order to familiarise them with the newest discoveries in their profession. Medical Director John C. Wise is the president of the naval medical school. He is a native of Virginia and entered the navy in 1870. He has seen much sea service and was fleet surgeon on the Baltimore during the Spanish war.

